# Provider knowledge and performance in medication injection safety in anesthesia: A mixed method prospective crosses sectional study

**DOI:** 10.1371/journal.pone.0207572

**Published:** 2018-12-05

**Authors:** Hasan Yusefzadeh, Alireza Didarloo, Bahram Nabilou

**Affiliations:** 1 Department of Public Health, School of Public Health, Urmia University of Medical Sciences, Urmia, Iran; 2 Department of Public Health, Social determinants of health Research Center, School of Public Health, Urmia University of Medical Sciences, Urmia, Iran; 3 Department of Public Health, Patient Safety Research Center, School of Public Health, Urmia University of Medical Sciences, Urmia, Iran; University of Minnesota Twin Cities, UNITED STATES

## Abstract

**Objectives:**

Injection safety during anesthesia is a challenging health care issue in Iranian hospitals. Anesthesia is one of the most medication-intensive procedures in healthcare and injecting patients are an integral part of that care. The present study aimed to assess the status of medication injection safety practice in a teaching center.

**Design, setting, participants:**

A prospective cross-sectional study was done in 2014–2015 at a 500-bed secondary level teaching hospital affiliated with Urmia University of Medical Sciences, Iran. The study population included providers of anesthesia in two groups of operating rooms (ORs) with different types of surgeries at the center. Data were collected using valid and reliable observation and a questionnaire instruments in two consecutive phases. Mann-Whitney U, Kruskal-Wallis, and Spearman correlation tests were used for data analyses.

**Results:**

A total of 345 injections were observed and recorded during the study period, 53% in group A ORs, and 47% in group B ORs. Eighty-two questionnaires were completed (96.5% response rate) to determine hospital injection practices and personal knowledge of injection safety. Adherence to safety requirements was observed in 58.5% of injections. Fifty five percent of respondents knew that hepatitis B, C, and HIV are blood borne diseases. Observed compliance with injection safety requirements was determined significant by OR groups (P = 0.00). Correlation was significant between observed injection safety practices by age and work experience (P = 0.00). The Kruskal-Wallis test showed a significant difference (P = 0.000) in observed safe injection practices among four job groups but not in reported adherence. Knowledge of respondents was significant by job groups about blood borne diseases and receiving three doses of hepatitis B vaccine.

**Conclusions:**

The study revealed that some of safe injection procedures were well carried out in our ORs, but that others were not. The reported adherence of staff was acceptable but their actual practices were unsafe. It is suggested to implement audits, provide safer supplies, and complete Hepatitis B vaccination of injection providers.

## 1. Introduction

Safety in the workplace is an essential characteristic of occupational health measures [1]. Health care organizations should establish a safety culture that requires all care processes and human resources to concentrate on improving the safety of care provision [2].

The aim of medical interventions is to save lives and to promote health; therefore Health Care Workers (HCWs) have a responsibility to prevent transmission of health-care related infection. Observance of safe injection practices and associated infection control is important for protecting patients and HCWs [3].

Inappropriate injection practices have adverse effects and can potentially cause harm to many patients from blood borne pathogens and secondary infections, hepatitis B, C and epidural abscesses [4]. Studies in health workforce in developing countries demonstrate insufficient knowledge of hepatitis B virus infection and poor compliance with measures to prevent diseases [5–8].

The operating room (OR), as the site for anesthetic procedures, is one of the most medication-intensive areas in hospitals. Anesthesia providers use more drugs, particularly high-alert drugs, than any other units but they operate with fewer safety standards [9]. OR employees including anesthesia providers face a higher risk of infection from blood-borne pathogens than HCWs in other areas [6, 10]. This group of HCWs does not always follow standard precautions and do not report every injury [10].

Unsafe injection practice by team members administering anesthesia can include transmission of diseases, improper use of single-dose or multi-dose vials, and inappropriate use or reuse of infusion sets, syringes, and needles [11]. Processes of prescribing, mixing, relabeling and administering medication are reported to be done in anesthesia practice without proper observation of safety requirements [12]. Also, these processes can cause error in a crowded and stressful ORs environment [9].

Medication error is a frequent cause of adverse events in anesthesia and various factors contribute to increased likelihood of medication error in the anesthesia process. Among these factors are; use of potent medication, the complex and special environment of an OR and multiple responsibilities of the anesthesiologist in patient management [13].

Studies conducted in Iran have revealed low levels of knowledge and adherence to safe injection practice, and high occurrence of Needle Stick Injuries (NSIs) [14–16]. Other studies have also indicated unsafe injection practices [17, 18] and higher rates of NSIs in OR [19]. Furthermore, some scholars have reported less than optimal adherence to standard precautions in Iran [20, 21]. In addition, safety culture has been reported to be absent or weak in Iranian hospitals [22, 23] and medical education [23].

In summary, there has been little research on the status of medication injection safety in anesthesia in Iran. Administration of injections is an inevitable part of the anesthesia process including induction, maintenance and termination so the purpose of this study was to identify the status of injection safety practices by anesthesia providers in a teaching center.

## 2. Method

This cross-sectional prospective study was done during 2014–2015 in a 500-bed capacity teaching hospital affiliated with Urmia University of Medical Sciences (UUMS). The elected hospital is the largest referring and teaching hospital among the seven teaching centers in West Azerbaijan province with 3,365,000 populations in the northwest of Iran. Hospitals affiliated to Medical Sciences Universities are the largest group of hospitals in health sector of Iran.

The study employed a mixed-method approach of observations of injections in ORs and a survey of injection providers about adherence to safe injection practices at the hospital. ORs were located in two floors of hospital named as ORs group A and ORs group B. The type of surgeries was different in the two ORs groups. General surgery and Eye surgeries were performed in the ORs of group A. Neurosurgery, orthopedics, ENT and urology were performed in the ORs of group B. Injections were provided by anesthesia team members, including student nurse anesthetists, nurse anesthetists, anesthesiology residents and attending anesthesiologists (N = 85). The study participants were assigned to either group A or group B ORs, and did not rotate between group A or B for the period of the study.

Inclusion criteria were; having direct contact with patients; more than two years work experience, working in the morning work shift, and being an anesthesia trainee (resident or nurse) with at least 3 semesters of training in the hospital. Exclusion criteria were: working on evening or night shift and unwillingness to participate in the study.

### 2.1. Instrument

Data were collected using a valid and reliable instrument (Sections Two and Five of ‘WHO tool C’, adjusted by research team) [24] in three parts as follows: 1) background and demographic information (age, gender, educational certificate, job, work experience, and ORs group A or group B), 2) an observation checklist, used to collect data regarding actual injection practices, and 3) a questionnaire to collect data regarding adherence to safe injection practices at the hospital and the individual’s knowledge about injection safety in ORs. The questionnaire consisted of 16 questions: 11 about adherence to safe injection practices at the hospital, 2 regarding the availability of training vaccination against hepatitis B, and 3 questions to assess knowledge about diseases transmitted by injections.

Thirteen items of observation checklist were rated as correct, incorrect and not applicable/not observed. The three remaining items of checklist were rated as follows: “using a new pair of gloves for each injection”; (new gloves used—gloves not changed- no gloves used), “recapping a used needle and syringe”; (with one hand- with two hands- not recapped) and “type of syringe used for the observed injection”; (standard disposable- auto-disable–sterilizable).

For the 11 items about adherence to safe practices, respondents were asked to grade injection practices on a five point Likert scale (always, usually, sometimes, rarely and never), in order to assess staff adherence to safe injection practices, their knowledge about required practices and, finally, the impact of supply shortages in ORs in the last 6 months. Two items were graded yes/no to assess availability of medication safety training and vaccination against hepatitis B. The three remaining items assessed the respondent’s knowledge about diseases transmitted by unsafe injection practices, number of Hepatitis B vaccine doses received and number of NSIs or sharps injuries.

The original questionnaire was translated into Persian using the 'forward-backward' method. Then another bilingual translator blind to the original questionnaire, back-translated the revised Persian version to its original language. Finally, the backward translation was compared with the original version and revisions were made where necessary.

Face and content validity evaluations of the translated questionnaire were determined by a team of two anesthesiologists and two safety experts. The content validity index (CVI) and the content validity ratio (CVR) were calculated 0.84 and 0.99, respectively. Items were deleted with CVI and CVR less than defined values. Internal consistency of the questionnaire was evaluated by Cronbach's α-coefficient (α = 0.715) in a pilot study.

### 2.2. Data collection procedure

Data collection was conducted in two consecutive phases. First, observations were conducted of actual injection practices over a sixth-month period. In the next phage a survey was conducted about injection practices as well as knowledge about regulations and risks of unsafe practices in ORs.

#### 2.2.1. Observation of injections

Two trainers of student nurse anesthetists were assigned to observe and record data regarding injection practices in ORs. In order to avoid awareness of the observation activity, selection criteria of the observers was that they had a daily routine of entering and exiting to the ORs. Observers recorded an observation checklist for each injection. Observation in ORs included the complete injection process, including preparation of drug, injection and disposal of needle.

#### 2.2.2. Questionnaire

In order to ensure that provider's actions during injections were a true reflection of their normal practice, and not influenced by the survey, questionnaires were presented after completion of the observational phase of the study. Questionnaires were distributed among anesthesia providers (N = 85) in the two groups of ORs and were asked to complete and return the questionnaires. Items on questionnaires were about typical safe injection practices at the hospital. The researchers also interviewed ORs supervisors for additional information.

### 2.3. Data analyses

SPSS (version 21.0 SPSS Inc., Chicago, Illinois, USA) was used to analyze collected data. Results were expressed as descriptive (frequencies, ratios, percentages and means) and analytical statistics. The Kolmogorov–Smirnov test was used to investigate normality of the dependent variables distribution. The Mann Whitney U was used to compare the safe injection requirements significance by gender and OR groups. Kruskal-wallis test was used to evaluate statistical differences in observed and reported adherence to safe injection practices and knowledge among four job groups.

Associations between observed and reported injection safety practices and knowledge of providers with OR groups and gender were conducted by Spearman correlation coefficient. Association between observed injection safety practices and work experience was conducted by Pearson correlation coefficient. The significance level of results was set at P <0.05.

The STROBE checklists were used as reporting guideline. The study was approved by ethics committee at UUMS (No: IR.UMSU.1392.237). Completion of questionnaires was voluntary and injection providers announced their agreement in writing. Informed consent obtained from all patients before anesthesia at the patient entrance zone of the operating rooms verbally. Adult patients themselves declared their consent. In regard unconscious cases and minors, verbal consent obtained from their guardians and parents, respectively.

## 3. Results

During the study period, 345 individual injections were observed (53% in group A ORs, 47% in group B ORs) to evaluate adherence to injection safety practices. Nurse Anesthetists performed 45.5% (157/345) of observed injections. Eighty-two questionnaires were completed and returned (96.5% response rate) by providers regarding local adherence to and knowledge of injection safety. Demographic data of providers is shown in Table 1.

**Table 1:** Demographic and background characteristics of anesthesia injection providers (N = 82).

No.	Variable		N	%
**1.**	**Age group**	<30	36	43.9
**1.**		31–40	31	37.8
**1.**		41–50	14	17.1
**1.**		>50	1	1.2
**2.**	**Sex**	Male	44	53.6
**1.**		Female	38	46.4
**3.**	**Work experience** **(year)**	<10	47	57.3
**1.**		10–20	19	23.2
**1.**		>20	16	19.5
**4.**	**Number of staff**	ORs A	35	42.7
**1.**		ORs B	47	57.3
**5.**	**Job group**	Student	29	35
**1.**		Nurse	35	43
**1.**		Resident	12	15
**1.**		Attending	6	7
**6.**	**Training on injection safety**	Trained	59	72
**1.**		Untrained	23	18
**7.**	**Vaccination against Hep. B**	Received	68	85
**1.**		Not received	14	15

### 3.1. Observation data

According to observation data, the highest compliance with safe injection requirements was observed in the items; “standard disposable syringe was used for the injection” (99% of injections) and “for this injection, a syringe and needle was taken from a sterile unopened packet” (98% of injections).

The lowest compliance with safe injection requirements was observed for the items; “reconstitution of a medicine performed using diluents from the same manufacturer” at 17% and hand hygiene, with only injection providers washing their hands only 19% of observed injections, and using alcohol-based hand rub only 15% of the time (Table 2). Overall, safety requirements were observed in 61.28% of injections with considerable variation in values of items (minimum = 15.4, maximum = 99.4, Standard Deviation = 33.58).

**Table 2:** Frequency and percentages of observed injection practices in ORs.

NO	Item observed	Yes		No		N
		No.	%	No.	%	
**1**	**Was the injection prepared on a visibly clean, dedicated table or tray?**	314	98.4	5	1.6	319
**2**	**Did the provider wash her/his hands before preparing an injection with soap and running water?**	62	19	267	81	329
**3**	**Did the provider cleanse her/his hands before preparing an injection by using alcohol-based hand rub?**	51	15.4	281	84.6	332
**4**	**Standard disposable syringe was used for the injection.**	343	99.4	2	0.6	345
**5**	**For this injection, was a syringe and needle taken from a sterile unopened packet?**	338	99	3	1	341
**6**	**For reconstitution, were a syringe and needle each taken from a sterile unopened packet?**	316	98.7	4	1.3	320
**7**	**Reconstitution of a medicine performed using diluents from the same manufacturer**	18	17	90	83	108
**8**	**Using the correct volume of diluents in reconstitution of a medicine**	99	97	3	3	102
**9**	**Cleaning the rubber cap with antiseptic in case of a multi-dose vial**	41	56	32	44	73
**10**	**Cleaning the rubber cap with dirty swab in case of a multi-dose vial**	84	88.4	11	11.6	95
**11**	**Changing needle After withdrawing one dose**	37	52	34	48	71
**12**	**using a clean barrier to protect fingers when breaking the top from the glass ampoule**	126	43.5	132	56.5	258
**13**	**Keeping a temperature sensitive medication vial between 2°- 8°C during the period of use**	50	70.4	21	29.6	71
**14**	**Did the provider use a new pair of gloves?**	27	12.5	190	87.5	217
**15**	**Did the provider recap the used needle and syringe?**	249	72.2	96	27.8	345
**16**	**Was a needle-remover or needle destroyer used?**	107	41.6	150	58.4	257

### 3.2. Reported adherence to safe injection practices

On average, 80.3% and 79.7% of questionnaire respondents reported that sterilizable needles and syringes had never been used in their hospital for injections and for blood/fluid sampling, respectively. Needle removers or needle destroyers were used in only half (44%) of injections. Thirteen percent of respondents had never reported NSIs or sharps injuries to a person in charge and 17.1% of them sometimes reported NSIs or sharp injuries.

The teaching center had provided the necessary supplies for injections in 88% of cases. Twenty four percent of respondents reported that there had been stock-outs of puncture resistant sharps’ containers “sometimes” during the past six months.

Guidelines outlining post-exposure management procedures were available in the ORs “always or often” from the viewpoint of 53.7% of respondents (n = 44). Forty two percent of respondents (n = 35) confirmed availability of training regarding injection safety within the last two years. Supporting and counseling for blood and body fluid exposure, “always or often” was reported by 55% of respondents (n = 45) (Table 3).

**Table 3:** Frequency of reported adherence to injection safety practices.

NO	Items of adherence to injection safety practices	Always	Often	Usually	Some-times	never
**1**	**Using sterilizable needles and syringes to administer injections**	00.0	1.4	6.1	12.2	80.3
**2**	**Using sterilizable needles and syringes during blood or other fluid sampling**	00.0	2	2.4	15.9	79.7
**3**	**Using sterile equipment during performance of injections or infusions**	85.3	11.0	3.7	00.0	00.0
**4**	**Using a needle remover or needle destroyer**	20.5	11.5	12	11.1	44.9
**5**	**Reporting the injury to person in charge (If NSIs or sharps injury occurred)**	29.3	26.8	11.0	17.1	13.4
**6**	**Bringing injection devices for a therapeutic injection by patients**	1.2.1	00.0	3.7	6.2	88.8
**7**	**stock-outs of puncture resistant sharps containers during**	16.1	8.5	3.7	34.1	37.6
**8**	**Availability of guidelines outlining post-exposure management procedures**	32.4	22.5	13.4	14.6	17.1
**9**	**Supporting and counseling the staff for blood and body fluid exposures**	19.7	35.9	17.1	16.2	11.1
**10**	**Offering counseling (If you reported NSIs or sharps injury)**	29	25	9.8	12.2	22.0
**11**	**Offering disease testing (If you reported NSIs or sharps injury)**	37.2	23.7	15.9	9.8	13.4

Accidents from NSIs or sharps injuries were reported by 45% of providers (n = 37). Such incidences had occurred in 18.2% of providers (n = 15) once and 12.4% of them (n = 10) twice in the last six months. 85% of respondents (n = 72) had received at least one dose of hepatitis B vaccines.

**Fig 1:**
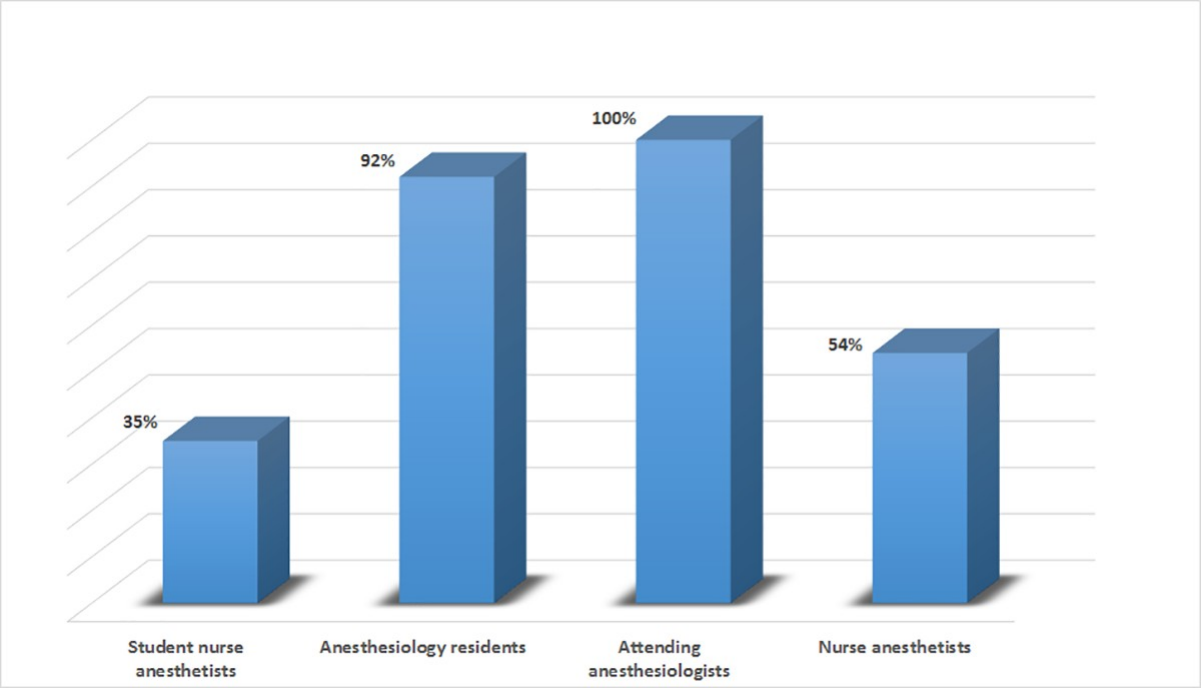
Percentages of injection providers who correctly knew that HIV, hepatitis B and C can all be transmitted by unsafe injection practices.

Only 55% percent of respondents (n = 46) knew that hepatitis B, C, and HIV are blood borne diseases, and only 26% of respondents (n = 22) believed that only hepatitis B can be transmitted by injection (Fig 1).

**Fig 2:**
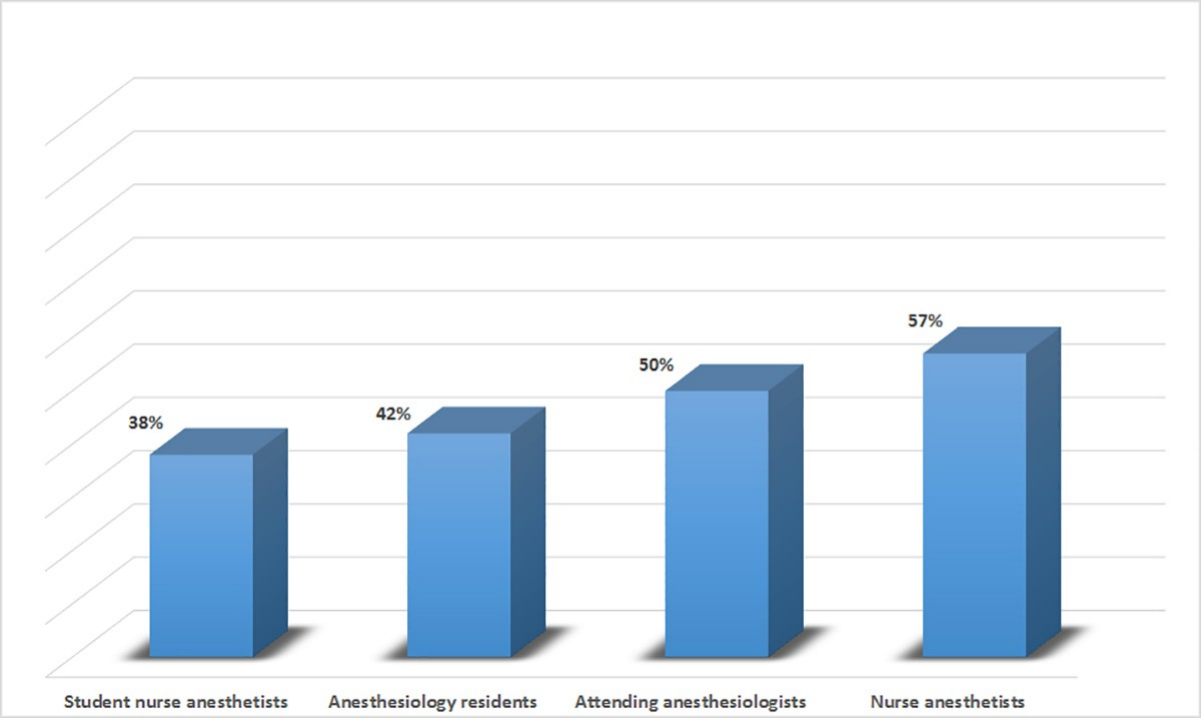
Percentages of injection providers who had received three doses of hepatitis B vaccine.

Forty eight percent of respondents (n = 39) had received three dose of hepatitis B vaccine (Fig 2).

### 3.3. Observational data analysis

Regarding observation data analysis, the Mann Whitney U showed that compliance with safe injection practices was not significantly different by gender (P = 0.276) but was different between OR groups A and B (P = 0.00). Mean score of observation in ORs B (m = 3.67, 95%CI = 3.19–4.15) was higher than ORs A (m = 1.74, 95%CI = 1.33–2.16). Work experience was significantly different between OR groups, with mean score of work experience in ORs A (m = 5.54, 95%CI = 4.63–6.44) significantly less than ORs B (m = 8.25, 95%CI = 7.26–9.24). Pearson correlation test showed significant correlation between observed injection safety practices, with age (P = 0.00, r = 0.208) and work experience (P = .00, r = 0.193).

The Kruskal-Wallis showed significant difference (P = 0.00) in safe injection practices among the four job groups involved in administration of injections, with a higher rate of injection safety practices among attending anesthesiologists that the three other job groups. Attending anesthesiologists and student nurse anesthetists obtained the highest (m = 5.52, 95%CI = 4.40–6.64) and lowest (m = 0.58, 95%CI = 0.07–1.24) mean scores in safe injection practices, respectively.

### 3.4. Reported adherence analysis

There were no significant differences between gender or OR groups for reported adherence to safe injection practices. In addition, there was no significant difference in responses among the four job groups.

Knowledge of respondents were significant by four job groups about diseases transmitted by injections (p = 0.003) and receiving 3 doses of Hepatitis B Vaccine (P = 0.001). There were no significant differences between gender or OR groups for knowledge about safe injection practices.

## 4. Discussion

Patient safety is a relatively new field in Iran as patient-safety-friendly hospitals started in this country in 2010 [25]. Medical and paramedical staff are the most important performers of patient safety practices including injections [26]. This observational and questionnaire study showed that reported adherence of the staff to safe injection practices was acceptable but their actual practices were unsafe. Knowledge of providers was moderate about diseases transmitted by injections. The observed and reported adherence to safe injection practices was complementary in some respects.

The WHO developed the observational method and questionnaire [24], to help hospitals, health systems, and even nations determine where the practices are good and where they are not. This will help leaders develop educational and training programs, or audits to encourage compliance with safe injection practices and finally, safety culture.

Results of the Ford study in ORs showed that 22% of providers had used the same needle or syringe to withdraw medication from a multi-dose vial [11]. In this study, re-use of the needles were considerably higher. This remarkable difference may have been caused by a lack of attention to staff training and supervision, or regular audits. Another factor was frequency of withdrawal of medication for anesthesia maintenance.

In a study conducted by Abkar, recapping after injection was observed in 61% of injections and packed needles and syringes were used in 98.8% of observations [27]. Kaphle reported recapping in 94.1% of injections [28]. Omorogbe reported that 23.0% of respondents recapped used needles regularly and 32.8% of them recapped sometimes [29]. The rate of recapping were 58% in the studies of Vong and Paul [30, 31]. The relevant values were also high in this study and had Similarities and differences with the above-mentioned studies.

According to the WHO guidelines, the needle should be discarded after an injection without recapping [30, 32]. In anesthesia processes, there is probably a high recapping rate because of repeated use of previously prepared medication in the syringe during a short period of time, but this does not explain two hand recapping and suggests other factors such as workload and safety culture [25].

Paul, in his study reported that 12.5% of providers had washed their hands with soap and water before administering an injection; only 7/3% of nursing staff had used sterile gloves during injections [31], less than related results in our study. The necessity for hand hygiene in injections will differ depending on whether there was contact with soil, blood or body fluids. Single-use gloves may be indicated when performing intravenous device insertions and infusions [24]. This procedures are common in the ORs and Lower values for those can be attributed to a knowledge gap or an excessive workload [33, 34].

Results of a study by Gounder showed that not using a new needle and syringe for each new patient or to access medication vials was observed in 3% and 28% of cases, respectively [35]. In this regard results of the present study were acceptable. Not using a new needle could justify limiting factors such as excessive workload and shortage of a supply.

Maintaining an adequate supply of single-use devices is essential to enable providers to use new devices each time they perform a procedure. In our study providers declared stock-outs of some supplies during the last 6 months.

Giard reported a 94.5% compliance rate “always" by HCWs for changing gloves between patients [36]. Hutin declared factors of unsafe injection practice as; a shortage of single use injection devices, insufficient awareness of the risk of HIV infection from unsafe injections, and weakness in waste management sharps waste management [37]. In the present study providers used new gloves in 37% of injections.

Omorogbe reported that 58.2% of respondents had NSIs [29]. This amount included 53% of providers during the previous 12 months[30], similar to our rate. Less than half of our respondents knew that hepatitis B, C, and HIV can all be transmitted via unsafe injection, which is much lower than the rate reported by Vong, et al, who reported that 90% were aware of this risk [30, 31], indicating a knowledge gap.

In Omorogbe’s study knowledge was significantly influenced by gender, and work experience of nurses [29], different from our findings. In this regard observed compliance with requirements was better in ORs B. Work experience of providers was higher in ORs B which could be an effective factor.

Low rate of vaccination against Hepatitis B Virus (HBV) was reported by providers with different coverage between them. Reported rate of HBV vaccination coverage were 70.1%, and 73% for physicians, and nurses in Iran, respectively [38] different from our results in ORs. Batra reported that 49.6% of HCWs received HBV vaccine [39], similar to present study.

In our study reported knowledge of providers about blood borne diseases was also modest like their behaviors in receiving hepatitis B vaccine. Adherence to universal precautions, such as safe needle disposal and wearing gloves during phlebotomy are weak among HCWs in developing countries [40–41].

Lack of enough Knowledge about blood borne diseases could be the reason for not being vaccinated. Other possible reasons are workload or efficacy of HBV from the point of view of providers.

### 4.1 Strengths and limitations of this study

Our study is limited in that it is from a single teaching hospital, and so may not be generalizable to the rest of our system, or the rest of Iran. In addition, we studied what was done but not why, so cannot explain why group B adhered more closely to correct practices, while group A, having the same knowledge, did not actually use these practices. This study is valuable, however, in that there is very little information about the status of medication injection safety in anesthesia in Iran, and that, by using a mixed approach (observation and questionnaire), we could test both the knowledge about safe practices, and the actual implementation of safe practices.

## 5. Conclusions

The study revealed that in ORs at selected center, some of the WHO guidelines about safe injection procedures were well carried out (dedicated table for injections and use of sterile disposable syringe) while other safe injection practices were unsatisfactorily implemented (no recapping, no use of disposal bins for sharps and no receiving complete doses of Hepatitis B vaccinations among providers). The important point of the study was that the knowledge of staff was acceptable but their actual practices were unsafe.

It is suggested to implement audits for safe injections, provide safer supplies e.g. auto-disable syringes, and Hepatitis B vaccine with complete doses to all injection providers in ORs. Finally, it seems further study is needed to find probable system or human problems in this regard.

## Supporting information

S1 Dataset (SAV)

S1 Appendix (TIF)
